# The New Anæsthetic

**Published:** 1857-03

**Authors:** 


*The New Anæsthetic.*

Our London exchanges contain accounts of experiments by Dr. John Snow, on the use of the vapor of amylene, by which it appears that this substance possesses anæsthetic properties not inferior to chloroform and ether. Dr. Snow first tried the effect of this hitherto but little-known remedy upon some of the inferior animals, and witnessing no ill effects he took the vapor himself with a like result. Since then he has administered it to quite a number of patients in the London hospitals, with very good results. Mr. Fergusson, Mr. Bowman, and others, have allowed it to be employed in some of their operations, and express themselves as satisfied with its good effect in causing an entire prevention of pain, and an apparently pleasant sleep. Amylene is made by distilling fusel oil with chloride of zinc; its composition, is 10 atoms of hydrogen, and 10 of carbon; its specific gravity is somewhere in the neighborhood of .700; and its boiling point 102° Fahr. It was first discovered by Cahours, about fifteen years ago, but Dr. Snow has the entire credit of demonstrating its anæsthetic properties. Its relative advantages and disadvantages, as stated by Dr. Snow in the *Times and Gazette*, are as follows:—' In regard to its odor, it is more objectionable than chloroform, but much less so than sulphuric ether. In respect to its pungency, it has a great advantage over both ether and chloroform, being much less pungent than either of them. Thus, whilst the patient, especially if a female, often complains of a choking feeling and want of breath in commencing to inhale chloroform, and two or three minutes are lost before the vapor can be inhaled in any useful quantity, she can begin to inhale the amylene in full strength within half a minute from commencing, and the operation may generally be begun within three minutes. In the amount which suffices to induce insensibility, it is intermediate between chloroform and ether, chloroform having the advantage. Amylene has an advantage in preventing pain with a less profound stupor than that occasioned by the other agents; and, in the ready waking and recovery of the patient, it has an advantage over chloroform, and a still greater advantage over ether. Its probable safety I have spoken of; and the greatest advantage of all, if it should continue to be met with in all cases, is the absence of sickness from its use. The almost entire absence of struggling and rigidity may also be mentioned as an advantage of amylene over ether and chloroform.'—*North Am. Medico-Chirurgical Review.*

---

☞ Gustav. C. E. Weber, M.D. of New York, has been appointed Professor of Surgery in the Cleveland Medical College, in place of Prof. Ackley resigned.—*Ibid.*

---

☞ Dr. J. Aitken Meigs has been appointed Professor of the Institutes in the Philadelphia College of Medicine, Dr. H. Hartshorne having been transferred to the Chair of Practice. Dr. W. Hembel Taggart has also been elected Professor of Materia Medica in the same College, in place of Dr. Tyson, resigned.—*Ibid.*

